# HEROIC: a platform for remote collection of electroencephalographic data using consumer-grade brain wearables

**DOI:** 10.1186/s12859-024-05865-9

**Published:** 2024-07-18

**Authors:** Richard James Sugden, Ingrid Campbell, Viet-Linh Luke Pham-Kim-Nghiem-Phu, Randa Higazy, Eliza Dent, Kim Edelstein, Alberto Leon, Phedias Diamandis

**Affiliations:** 1 Department of Medical Biophysics, University of Toronto, Toronto, ON M5S 1A8 Canada; 2 Princess Margaret Cancer Center, University Health Network, 610 University Avenue, Toronto, ON M5G 2C1 Canada; 3 Department of Laboratory Medicine and Pathobiology, University of Toronto, Toronto, ON M5S 1A8 Canada; 4 Laboratory Medicine Program, University Health Network, 200 Elizabeth Street, Toronto, ON M5G 2C4 Canada; 5 Cognitive Science Program, McGill University, 845 Rue Sherbrooke O, Montréal, QC H3A 0G4 Canada; 6 Department of Supportive Care, Princess Margaret Cancer Centre, Toronto, ON M5G 2C4 Canada; 7 Department of Psychiatry, University of Toronto, Toronto, ON M5S 1A8 Canada; 8 Department of Pathology, University Health Network 12-308, Toronto Medical Discovery Tower (TMDT), 101 College St, Toronto, M5G 1L7 Canada

**Keywords:** Electroencephalography, Wearable devices, Remote medicine

## Abstract

The growing number of portable consumer-grade electroencephalography (EEG) wearables offers potential to track brain activity and neurological disease in real-world environments. However, accompanying open software tools to standardize custom recordings and help guide independent operation by users is lacking. To address this gap, we developed HEROIC, an open-source software that allows participants to remotely collect advanced EEG data without the aid of an expert technician. The aim of HEROIC is to provide an open software platform that can be coupled with consumer grade wearables to record EEG data during customized neurocognitive tasks outside of traditional research environments. This article contains a description of HEROIC’s implementation, how it can be used by researchers and a proof-of-concept demonstration highlighting the potential for HEROIC to be used as a scalable and low-cost EEG data collection tool. Specifically, we used HEROIC to guide healthy participants through standardized neurocognitive tasks and captured complex brain data including event-related potentials (ERPs) and powerband changes in participants’ homes. Our results demonstrate HEROIC’s capability to generate data precisely synchronized to presented stimuli, using a low-cost, remote protocol without reliance on an expert operator to administer sessions. Together, our software and its capabilities provide the first democratized and scalable platform for large-scale remote and longitudinal analysis of brain health and disease.

**Supplementary Information:**

The online version contains supplementary material available at 10.1186/s12859-024-05865-9.

## Introduction

The recent emergence of consumer-grade wearable electroencephalographic (EEG) systems has opened potential new avenues for research and investigation into the understanding of the human brain through postnatal development [1] and neurological disease [2, 3]. Unlike traditional EEG devices that are largely confined to hospitals and research settings, brain wearables are portable, battery-operated, and require just a few minutes for setup without the need for a modality-specific technician [4]. Because of these practical advantages, coupled with significantly lower cost (~ $200–900 USD/device [5]), there is hope that these devices may facilitate detection and monitoring of neurological pathologies outside of traditional centralized research and healthcare settings [6, 7]. Despite their accessibility and ease of use, the integration of such wearable devices into large-scale longitudinal research outside of clinical settings remains largely unexplored due to the absence of a suitable framework for standardized data collection and analysis.

Previous work using the four-electrode “Muse 2” EEG headbands has shown that these consumer-grade devices can capture key EEG analysis metrics, such as event-related potential (ERP) and resting state power spectral data that are comparable to clinical-grade EEG systems [8, 9]. Moreover, using three-minute recordings from the same devices, others have shown the ability to predict stroke severity by comparing the power spectral properties of ischemic stroke patients and healthy controls [10]. Other hardware like the Emotiv EPOC + Saline Flex have also been validated for the purposes of ERP collection using an approach involving simultaneous recordings with a research-grade system [11]. While these important pioneering studies demonstrated the potential utility of consumer devices for cognitive monitoring and biomedical applications, they were largely confined to the research setting and were not designed to leverage their unique portability and operator-free benefits.

Other recent studies have used remote data collection protocols to demonstrate the practicability of gathering frequent at-home data from participants. For example, one group deployed the Muse 2 device to collect daily remote data over 14 days to study mindfulness and found correlations with self-report mind wandering metrics [12]. More recently, *Sidelinger *et al. validated remote and longitudinal spectral resting state EEG data from the Muse 2 by collecting data with a proprietary software. When they compared the wearable data to in-lab medical grade EEG recordings, they found significant correlation between self-reported trait anxiety and day-to-day variability of an individual’s alpha frequency [13]. The most popular application of at-home EEG are those designed to mimic sleep studies with polysomnography. For example, one group used connected an ear-EEG sensor to a portable amplifier, but required a technician to visit the users home [14]. Similarly, another study developed an at-home sleep-staging and apnea-detecting device, but also equipped the device in the presence of a technician [15]. These innovative works overcame the barriers associated with remote EEG collection, but there remains a clear need for an open-source system to allow participants to equip the devices independently and use them to carry out advanced neurocognitive assessments.

There have been various attempts to develop platforms for remote data collection, each with a different design focus. An early system known as “NeuroMonitor” allowed a small circuit board and wired electrodes to transmit data with Bluetooth [16]. Li et al. developed a similar portable EEG system that suppressed external noise sources and collected robust signal but still involved a circuit board being strapped to the user’s arm, prohibiting independent use by a patient population [17]. An open-source project known as “cEEGrid” developed a small wearable device that leveraged OpenBCI EEG signal acquisition platform, which is not adaptable to other commercially available hardware [18]. *Milne-Ives *et al*.* reviewed the subset of the available monitoring technologies for people with epilepsy, and found that few systems were both adequately reported and sufficiently capable [19]. One popular mobile application known as “Mind Monitor” has been used in many publications but this software is limited to the Muse devices and does not allow stimuli to be synchronized [20, 21]. Other commercial software like Interaxon’s “Muse Direct” suffers from a lack of synchronized event markers and others like “Emotiv Pro” have expensive subscription models [22, 23].

Notwithstanding these important milestones, there is still a clear need for an open-source companion platform to allow for implementation of cognitive tasks for the measurement of event-related potentials (ERPs) in the remote setting.^6^ Because ERPs have been shown to be informative for a variety of pathologies, the ability to monitor them remotely has important implications for the scalability of neurocognitive disease research. The current lack of a suitable platform for these purposes prohibits the deployment of EEG wearables toward novel avenues for the real-time and longitudinal study of disease detection and evolution. To address this critical gap, we have developed “HEROIC” (Home EEG Recording frOm Interfacing Computer), an open-source research platform capable of leveraging consumer-grade EEG wearables to longitudinally deploy a battery of cognitive process tasks to measure brain activity (including ERPs [9, 24–26]) in both research and remote settings. As a proof-of-concept, we deployed our system to record four at-home sessions from 14 healthy participants resulting in a dataset containing approximately 60 independent EEG recording sessions. We use these data to highlight that HEROIC is easy-to-use and reliable for collecting high quality data and support its ability to be deployed remotely and longitudinally to measure brain activity including complex markers like ERPs. We make our unique software and sample dataset publicly available to provide a democratized platform for the research community to use for the investigation of brain health and disease.

In addition to a brief introduction of the HEROIC platform, we describe its implementation and how it can be operated to collect EEG data. We also describe a proof-of-concept pilot study as an example of how a researcher could design a protocol that uses HEROIC. Finally, we will present the results and analysis of this feasibility study demonstrating that HEROIC is capable of remotely and independently measuring precise quantitative brain activity.

## Implementation

**Fig. 1:**
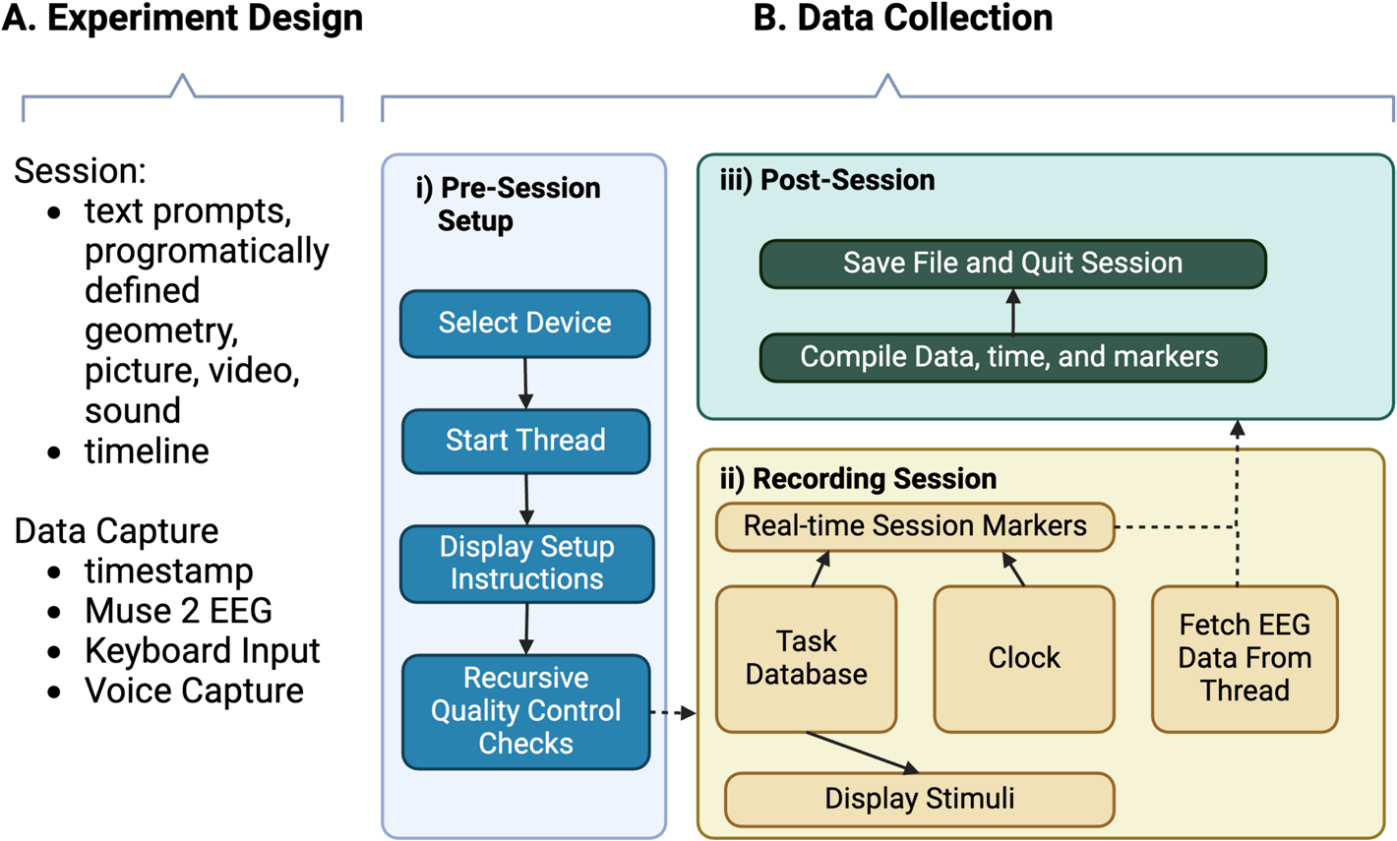
Schematic Overview of HEROIC Implementation.** A** Experiment design can draw from multiple different modalities of stimuli and data capture. **B** **i)** The choice of device is hard-coded but modifiable with subsequent modules reacting accordingly. **ii)** In a data collection session, the stimuli on the screen are determined by a configuration file in a local database of different tasks (e.g. eyes-open rest). The stimuli are marked with a signature number which are timestamped and overlaid onto the EEG data being read from the thread. **iii)** When the user has completed the session, it returns to the graphical user interface (GUI) where the user is prompted to close the program, thereby triggering the saving of the data files

We designed HEROIC with sufficient modularity such that the main components of test administration are customizable without affecting the core functionality of collecting data. For example, when designing an experiment, HEROIC sessions can include multiple modalities such as text, images, videos, and sound to help guide users through specific tasks. Similarly, data recording is agnostic to the modality (wearable device, keyboard input, voice capture etc.) (Fig. 1A). Overall, the execution of the data collection can be conceptualized as having three core stages: pre-session setup, a recording session, and a post-session completion step as discussed below (Fig. 1B).

### Device initialization and setup

**Fig. 2:**
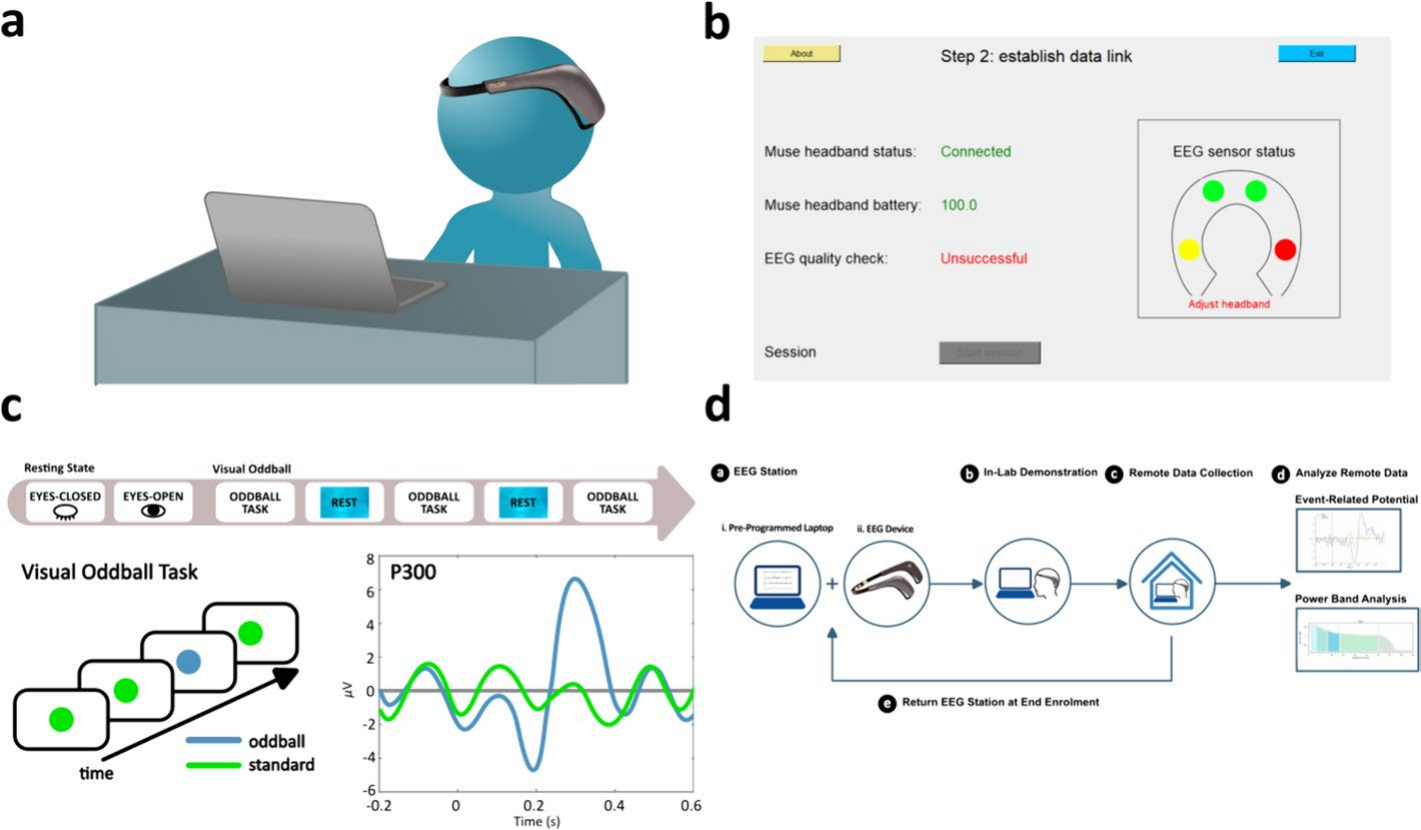
HEROIC: A lightweight software for remote collection of EEG data.** A** Diagram showing a participant wearing the Muse 2 EEG wearable while seated across a portable pre-programmed laptop with HEROIC. **B** Screenshot of HEROIC’s graphical user interface for its interactive initial signal quality check process. Colored circles represent the status of each electrode (Green: Accepted, low signal variability. Yellow: Close to Accepted. Red: Not Accepted, high signal variability). **C** Top: Sequence of cognitive process tasks employed in our default session design. Bottom Left: Diagram of visual oddball paradigm where oddball and standard stimuli are presented. Bottom Right: Diagram sample of a typical P300 waveform evoked by the oddball stimuli. **D** Standard workflow for remote data collection using the presented system. First, a laptop with software (a (i)) that can connect to and receive data from an EEG wearable (a (ii)) is provided to users. Participant attends an initial in-lab demonstration of the software to simultaneously perform EEG recordings with cognitive tests (b) of interest. Longitudinal data collection is done at home (c) with sessions as frequent as for example, weekly, bidaily, or even daily. Once collection is complete, or during regular hospital visits, devices are returned to the lab for data retrieval and analysis (d)

The core objective of the pre-session initialization setup step is to enable a participant to independently connect an EEG wearable to a receiver laptop and carry out a pre-recording quality control step (Fig. 2A). While multiple popular wearable EEG devices are hard coded options in the current version, researchers are also free to add additional devices as needed (Fig S1A). The detection of the selected device will trigger a sequence of events, starting with the opening of a computational thread for collecting that data. Next, the participant is guided through visual and written instructions on how to equip the selected device. And finally, they are taken to a page where they are given real-time feedback on the connection status and signal quality from each of the electrodes on the device (Fig. 2B). When the user has achieved good signal on all electrodes, they can click the button to begin the session.

### Session recording

This customizable portion of the HEROIC program is dedicated to guiding the user through different instructions to help standardize data collection. When the session begins, there is a hard-coded selection of a configuration file which contains a set of instructions allowing Python to generate the stimuli required for the session on the fly using media files or computationally defined stimuli (Fig S1B-C). For example, we provide a sample configuration of a session consisting of free recordings followed by oddball tasks interlaced with rest to allow the users to recover (Fig. 2C). While the session is running, data is timestamped in real-time, and every time a new stimulus is presented on the display, a designated numerical signature (e.g. oddball stimulus 2) is added to the marker column to allow for retrospective analysis of that event.

### Post-session completion

When the recording session is complete, the user is informed that the session has ended and they are then returned to the graphical user interface GUI, where they are prompted to close the program. The closing of the program prompts the compilation of the timestamped data with the stimuli markers into a comma separated value (CSV) file, which is then zipped with metadata files describing the configuration of the session, device used, etc. The closing of the program also terminates the thread and disconnects the device. The zipped files are finally saved to the hard drive in an output folder.

### Experiment design

Together, the modularity and capabilities of HEROIC allow for the development of novel study protocols. We provide a sample protocol where participants are brought to the research setting (e.g. hospital, research institute) for a one-time instruction on how to use HEROIC, and then they independently collect subsequent recordings at home. The computers and wearables are then returned to the researchers for analysis (Fig. 2D).

## Results

### HEROIC allows for reliable EEG data collection in remote settings

To assess the reliability of this software at collecting time-stamped EEG data in participants’ homes, we installed our software on ten modest (~ $300 USD) refurbished laptops and paired them with the Muse 2 EEG headset (~ $250 USD) (Fig. 2A). We deployed these HEROIC EEG stations to a cohort of 14 healthy participants from three Canadian cities (Toronto, Montreal, and London). Participants performed a brief 20-min demonstration on-site in a controlled research environment with lab personnel to learn how to connect and collect EEG data using the Muse 2 device paired with a HEROIC-installed laptop. Distinct from the other aforementioned studies, HEROIC was designed to also allow the capture of time-locked EEG data synchronized to the presentation of visual stimuli presented to users (i.e., an “oddball” task), to allow the collection of ERPs (Fig. 2C). To test the ability to remotely record EEG data, participants were then asked to take the hardware home and carry out a series of four additional EEG recording sessions within a nine-day period independent of any study operator (Fig. 2D).

**Fig. 3:**
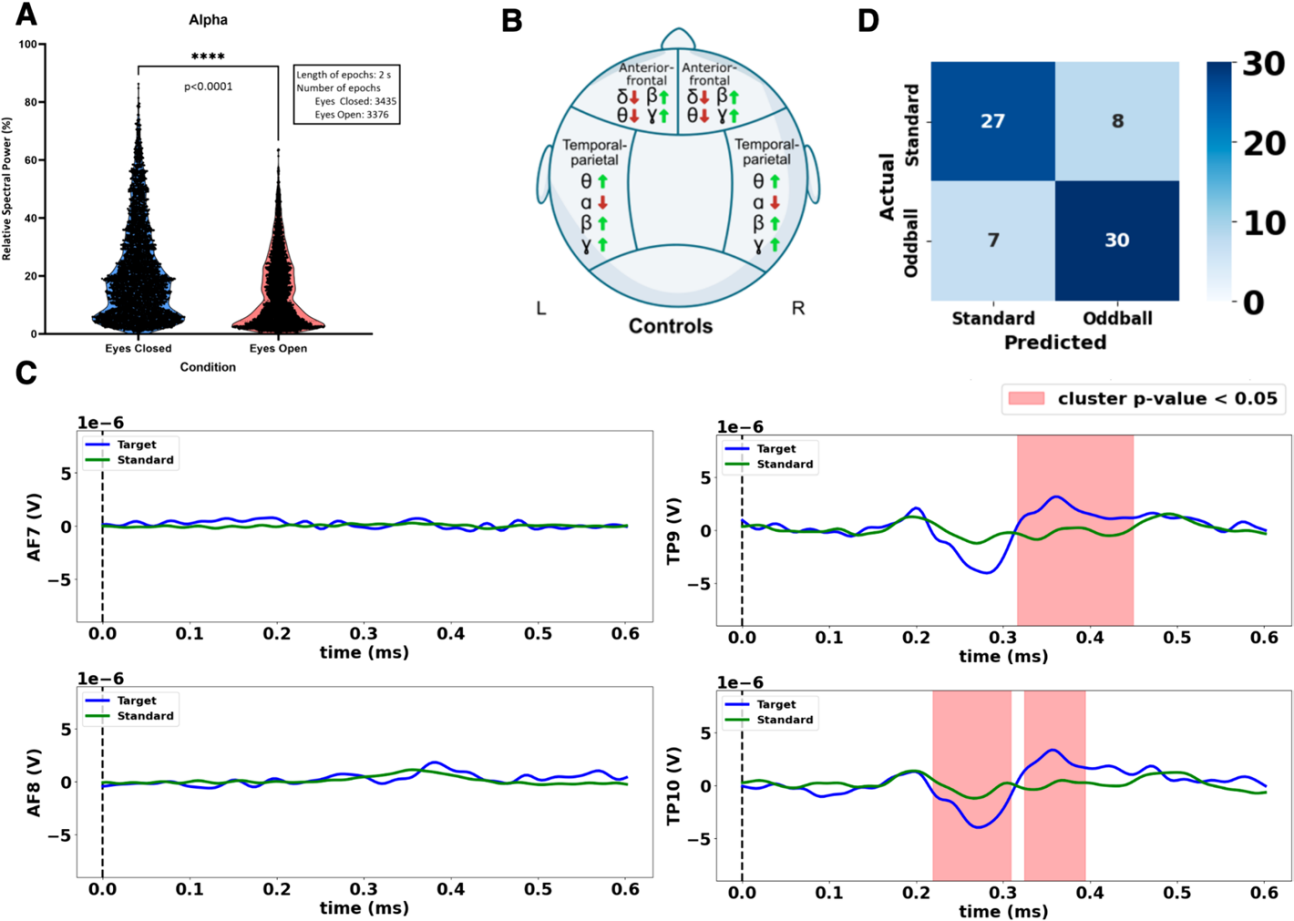
Remote detection of cognitive processes with portable EEG devices. **A** Alpha blocking: Paired violin plots showing a significant decrease in alpha activity between eyes-closed and eyes-open (p < 0.0001). Each point represents a two-second time event recorded from TP9 channel. **B** Spectral changes across scalp: Summarized significant changes in power spectral frequency bands changes from eyes-closed to eyes-open resting state conditions by brain regions in control participants, p-values shown in Supplemental Table 1. (δ delta, θ theta, α alpha, β beta, γ gamma). **C** P300 waveforms: Averaged ERP plots for each electrode derived from the combined data/ERPs of 14 healthy controls. Amplitude on y-axis in volts. Time on x-axis in seconds. Clustered t-tests detected significant (red, *p* < 0.01) differences between the oddball (blue) and standard (green) curves. Significant clusters resembling a P300 for TP9, and both a N200 and P300 for TP10 were detected. **D** Minimum-Distance-To-Means (MDM) Classifier: Single-epoch classifier performance (accuracy = 80.60%) confirms oddball and standard stimuli are reliably different

To assess the overall fidelity of HEROIC to reliably capture baseline EEG data, the platform guided participants through one-minute sessions of resting state in both open and closed eyes conditions. Acquisitions were referenced to the Fpz electrode position (10–20 International System) and was bandpass filtered between 1-55 Hz before analysis. Power band analysis confirmed that there was higher relative alpha power during periods of closed eyes (Fig. 3A; t-test *p* < 0.0001) as is well-documented in the literature [27]. Moreover, participants expressed expected symmetrical differences between open and closed eyes conditions on corresponding electrode positions on the left and right hemispheres (Fig. 3B). Together, these results suggest that HEROIC can remotely guide participants through multiple specific tasks and record annotated EEG data collected from an accessible consumer-grade device.

The precise time-stamping nature of our open software platform offers the exciting opportunity to carry out advanced cognitive processes measurement. One of the most well studied ERPs for measuring cognitive processes is the “P300” (positive deflection occurring approximately around 300 ms) which can be generated using the oddball task. We therefore also asked participants to perform a guided ERP visual-oddball task similar to that in [9] where which participants were shown a series of frequent “standard” stimuli (90% chance of green circle) and infrequent “oddball” stimuli (10% chance of blue circle). By time-stamping the recorded EEG data in HEROIC, we were able to observe distinct P300 waveforms in the TP9 and TP10 electrodes when the infrequent oddball stimuli were presented (permutation cluster t-tests TP9 N200: *p* = 0.001, TP9 P300: *p* = 0.001, TP10 N200: *p* = 0.173, P300: *p* = 0.001; Fig. 3C). Additionally, using a minimum-distance-to-means classifier, [28] we could successfully distinguish between individual oddball and standard stimuli epoch (accuracy: 0.8060; AUC: 0.8937; Chi square test: *p* < 0.0001) (Fig. 3D). Together, these results support that HEROIC can carry out fully automated time-stamped ERP experiments using low-cost EEG headsets even when carried out by participants independently at home.

## Discussion

Brain wearables have the potential to capture large volumes of EEG data outside of traditional research and healthcare environments. However, the lack of experienced operators, lower electrode densities, and precise time-stamping of data may limit current capabilities and promise. Due to these diverse factors, many early studies have focused on in-lab sessions and require averaging of data over large cohorts to get consistent results.^21–23^ While these works mark important initial milestones, the lack of a freely available platform for data recording still presents an important barrier that has likely limited deployment and progress to many exciting biomedical applications and remote care. Specifically, the literature lacks an open-source platform for the collection of complex EEG signatures, such as ERPs, in a way that is cost-effective and scalable over long timescales.

Here, we developed an open-source EEG platform that connects popular EEG devices to collect time-stamped data recordings and benchmarked the reliability across several relevant scenarios in healthy participants. Importantly, the presented platform can be run on modest refurbished laptops and can be easily customized and expanded to different EEG wearables and to administer additional automated cognitive tests. As a proof-of-concept, we deployed our platform to 14 healthy participants, which showed that we were able to recapitulate expected differences in resting states as well as reliably generate complex brain measurements like the P300 waveform. Given the relatively high adherence to the frequent recording protocol within our cohort, we believe this platform and protocol are easy to use and can be adapted to larger cohorts to study health and disease.

### Comparison with state-of-the-art

To properly contextualize the advancement made by HEROIC, we will compare our software to other common remote data collection applications. For example, the developers of the Muse 2 headband have released a freely available platform known as “Muse Direct” that allows for data collection using a laptop. This system has the added benefit that it can stream simultaneously from multiple people but it lacks the crucial ability to synchronize the data collection to the presentation of stimuli thereby prohibiting ERP analysis [22]. Another common data collection software is Emotiv Pro, which connects to Emotiv devices like the EPOC X. While this platform allows interactive quality checks and timestamped event markers similar to HEROIC, it requires expensive ongoing licensing fees (over $1000 per annum), which limits its scalability, especially in academic settings [23]. Together, these popular examples highlight the need for the combination of an open-source data collection platform with precise timestamped stimuli markers.

### Limitations and future directions

While HEROIC’s implementation is robust and ready for researchers to adopt for collecting their own data, it does have limitations which present opportunities for further development. For example, it is currently compatible with three popular EEG hardware systems (Muse 2, Muse S, and EPOC X) but new hardware systems are being released on an ongoing basis. We have intentionally made the core functionality agnostic of the hardware to allow future integration of these new hardware systems as they are released. Another limitation of our study is that our user feedback was collected in an open-ended manner with many iterative real-time refinements, prohibiting a meaningful structured analysis. Follow-up studies should include structured surveys with larger sample sizes to statistically power conclusions regarding the user experience and ease-of-use. We additionally note that our pilot study was only conducted on adults (> 18 years of age) and a separate pilot study would be needed to validate the use of HEROIC for monitoring younger populations. Finally, a major direction for future work is to establish that our system is usable in a sensitive patient population, and that the data is biomedically informative. Specifically, we envision that HEROIC would be used for a first-of-its-kind “massively serial” longitudinal study of brain health and disease, where patients with a neurological condition (e.g. a brain tumour) perform several (10 +) EEG recordings at home. Such a study, over a long enough period could provide the proof-of-concept for long-term tracking of brain states and if and how EEG patterns evolve with changes in neurological health.

## Conclusion

There are several logistical and technological barriers associated with the remote collection of EEG data using consumer-grade wearables. HEROIC addresses these limitations by providing a democratized, scalable, and customizable solution capable of collecting ERP measurements in remote settings without an expert operator. This platform has the potential to significantly advance our understanding of brain health and disease by enabling researchers to perform long-term longitudinal EEG studies at a low cost.

### Supplementary Information

Additional file1

## Data Availability

Project name: HEROIC. Project home page: https://github.com/diamandis-lab/HEROIC/tree/main. Operating systems: Windows 10. Programming language: Python. Other requirements: Python version 3.8.9, Bluetooth 4.0 or more recent. Installation guide has comprehensive list. License: GNU GPL. Restrictions for use by non-academics: None. The dataset supporting the conclusions of this article is available in the Zenodo repository, https://zenodo.org/records/11493897. The HEROIC software platform, installation instructions, and user guide can be found at https://github.com/diamandis-lab/HEROIC/tree/main.
