# Supplementary material for: HEROIC: a platform for remote collection of electroencephalographic data using consumer-grade brain wearables
Source: BMC Bioinformatics. 2024 Jul 18;25:243. doi: 10.1186/s12859-024-05865-9 (PMC11256487; doi:10.1186/s12859-024-05865-9)
Supplement: Supplementary file 1 — Additional file1 [file 12859_2024_5865_MOESM1_ESM.docx]

**Supplementary Figures**

**
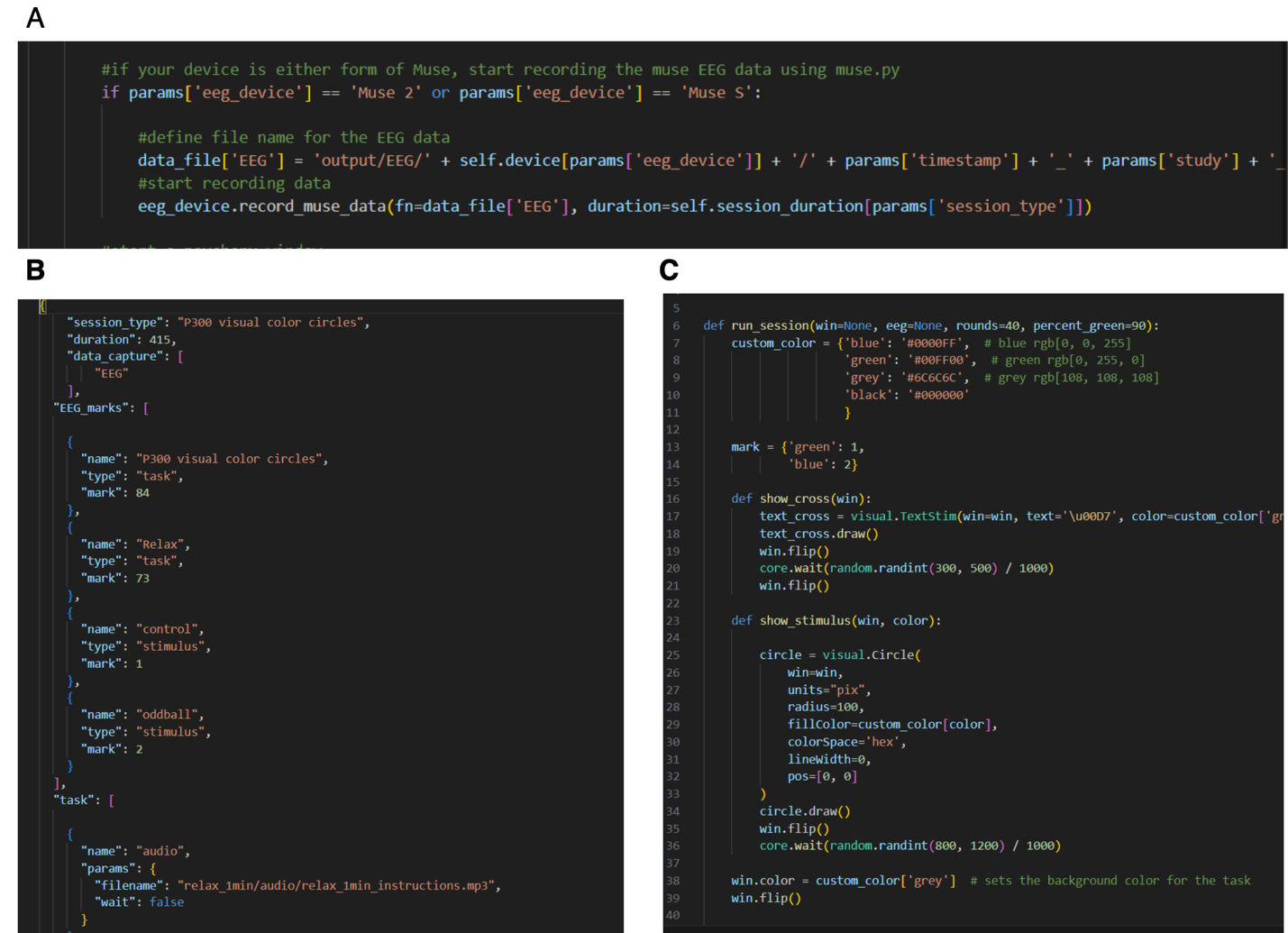
**

**Supplementary Figure 1| HEROIC is easily customizable. A.** Code sample showing the selection of the device leading to a specific function for collecting the data. **B.** Code sample showing the definition of stimuli for a visual oddball session, including markers and references to files to access media. **C.** Code sample showing the definition of a session configuration. Functions provide instructions on how to present the stimuli later as part of a sequence.

|  | **Temporal-parietal (TP)** | | **Anterior-frontal (AF)** | |
| --- | --- | --- | --- | --- |
| **Power band** | **TP9** | **TP10** | **AF7** | **AF8** |
| Delta, δ | 0.249 | 0.366 | ***1.15e-57 | ***2.23e -108 |
| Theta, θ | ***1.47e-58 | ***6.11e-51 | ***8.94e-15 | *** 4.01e-19 |
| Alpha, α | ***1.08e-66 | *** 1.45e-84 | 0.546 | 0.198 |
| Beta, β | ***2.05e-14 | ***3.62e-13 | ***1.56e-76 | ***1.66e-145 |
| Gamma, γ | ***6.56e-08 | ***3.521e-8 | ***6.82e-127 | ***2.98e-162 |

**Supplemental Table 1**| **Summary of symmetrical changes across scalp.** P-values by spectral changes across the scalp. Significant changes in power spectra frequency bands change from eyes-closed to eyes-open resting state conditions by brain regions in control participants. Overall, controls show symmetrical changes in brain activity between left (TP9) and right (TP10) temporal-parietal regions, and between left (AF7) and right (AF8) anterior-frontal regions.

*** p<0.0001, ns non significant, green boxes indicate an increase from eyes-closed to eyes open, red boxes indicate a decrease from eyes-closed to eyes-open.
